# Perilipin 2 Impacts Acute Kidney Injury via Regulation of PPAR*α*

**DOI:** 10.1155/2021/9972704

**Published:** 2021-09-09

**Authors:** Sujuan Xu, Edward Lee, Zhaoxing Sun, Xiaoyan Wang, Ting Ren, Zhouping Zou, Jifu Jin, Jie Li, Jian Zhang, Yingxiang Li, Qiang Yang, Yang Zhang, Man Guo, Yi Fang, Xiaoqiang Ding

**Affiliations:** ^1^Department of Nephrology, Zhongshan Hospital, Fudan University, Shanghai, China; ^2^Human Phenome Institute, Fudan University, 825 Zhangheng Road, Shanghai, China; ^3^Department of Cardiovascular Medicine, Shanghai East Hospital, Tongji University School of Medicine, Shanghai, China; ^4^Institutes of Biomedical Sciences, Fudan University, Shanghai, China; ^5^Shanghai Medical Center of Kidney Disease, China; ^6^Kidney and Dialysis Institute of Shanghai, China; ^7^Kidney and Blood Purification Key Laboratory of Shanghai, China; ^8^Hemodialysis Quality Control Center of Shanghai, Shanghai, China

## Abstract

Renal ischemia-reperfusion (I/R) can induce oxidative stress and injury via the generation of reactive oxygen species (ROS). Renal proximal tubular cells are susceptible to oxidative stress, and the dysregulation of renal proximal tubular cellular homeostasis can damage cells via apoptotic pathways. A recent study showed that the generation of ROS can increase perilipin 2 (Plin2) expression in HepG2 cells. Some evidence has also demonstrated the association between Plin2 expression and renal tumors. However, the underlying mechanism of Plin2 in I/R-induced acute kidney injury (AKI) remains elusive. Here, using a mouse model of I/R-induced AKI, we found that ROS generation was increased and the expression of Plin2 was significantly upregulated. An in vitro study further revealed that the expression of Plin2, and the generation of ROS were significantly upregulated in primary tubular cells treated with hydrogen peroxide. Accordingly, Plin2 knockdown decreased apoptosis in renal proximal tubular epithelial cells treated with hydrogen peroxide, which depended on the activation of peroxisome proliferator-activated receptor *α* (PPAR*α*). Overall, the present study demonstrated that Plin2 is involved in AKI; knockdown of this marker might limit apoptosis via the activation of PPAR*α*. Consequently, the downregulation of Plin2 could be a novel therapeutic strategy for AKI.

## 1. Introduction

Acute kidney injury (AKI) is a problem associated with rapid renal dysfunction and high mortality [1], which is often caused by renal ischemia-reperfusion (I/R) in clinics. Renal I/R injury (IRI) is characterized by the restriction of blood supply to the kidney followed by the restoration of blood flow. Currently, there are few therapies for IRI. I/R can induce oxidative stress and injure organs via the generation of reactive oxygen species (ROS). In IRI, the production of ROS remains high for 24 h after reperfusion [2]. It was found that renal proximal tubular cells are susceptible to this oxidative stress. The dysregulation of renal proximal tubular cellular homeostasis can damage cells via apoptotic pathways [3–5]. Therefore, managing ROS is an important target for the prevention and treatment of AKI.

The main cellular lipid droplet proteins are members of the perilipin family. There are five members of the perilipin family (Plin1–5), and these proteins have an amphipathic helical structure with large hydrophobic residues, which can bind tightly to the lipid droplet surface [6]. Each perilipin isoform has a different role, but few have been studied in the context of renal proximal tubular cells. Among them, perilipin 2 (Plin2) was the first lipid droplet surface protein to be identified, and it has been considered the marker protein of lipid droplets. Plin2 interacts with many signaling pathways. It also affects the homeostasis of intracellular lipid metabolism and promotes the accumulation of intracellular lipids by regulating the PPAR*α*-RXRA and CREB-CREBBP pathways. The transcriptional coactivator CREB binding protein (CREBBP) is important for the function of CREB, and the overexpression of Plin2 increases CREBBP expression, which promotes CREB transcriptional activity, consequently enhancing CREB functions [7]. PPAR*α* is a free fatty acid receptor that plays an important role in maintaining the homeostasis of lipid metabolism. Plin2 activation can protect neutral lipids from hydrolysis by lipases, affecting the expression and activation of PPAR*α* [7, 8]. Plin2 also regulates lipophagy in the heart [9]. Furthermore, elevated levels of ROS increase Plin2 expression and promote lipid droplet formation in HepG2 cells [7]. Some evidence has also demonstrated an association between Plin2 expression and renal tumors [10–12]. However, the role of Plin2 in IRI has not been investigated to date.

In the present study, we found that the expression of Plin2 was upregulated in the kidneys of mice after I/R treatment. Furthermore, mitochondrial ROS generation and apoptosis were associated with I/R. In vitro, hydrogen peroxide treatment was found to increase the expression of Plin2 and the generation of ROS in primary tubular cells. Moreover, Plin2 knockdown decreased apoptosis after hydrogen peroxide treatment, which was dependent on the activation of PPAR*α*. Collectively, we suggest that a Plin2 inhibitor may be a promising treatment for IRI, functioning through the inhibition of oxidative stress.

## 2. Materials and Methods

### 2.1. AKI Animal Model Induced by I/R

The animal experiments were approved by the Animal Care and Use of Committee of Zhongshan Hospital and performed in accordance with the National Institutes of Health Guide for the Care and Use of Laboratory Animals. Male C57BL/6 mice (8-10 weeks old) were obtained from the Animal Center of Jiesijie company. The I/R procedure was performed with bilateral renal pedicles clamped for 30 min, and the animal body temperature was maintained at 36-37°C. The sham group underwent the same process except the pedicles were not clamped [13].

### 2.2. Chemicals and Reagents

The anti-Plin2 antibody (NB110-40877) anti-PPAR*α* antibody (NBP2-76958) were obtained from NOVUS. The anti-Bax (14796), anti-Bcl-2 (3498), anti-pro-Caspase-3 (9662), and anti-cleaved-Caspase-3 (9664) antibodies were obtained from Cell Signaling Technology. Plin2 overexpression adenoviruses (Ad-Plin2), green fluorescent protein- (GFP-) expressing control adenoviruses (Ad-GFP), Plin2 knockdown adenoviruses, and control adenoviruses were obtained from Heyuan Biotechnology. The terminal deoxynucleotidyl transferase dUTP nick-end labeling (TUNEL) assay kit was purchased from Beyotime Biotechnology.

### 2.3. Culture of Primary Mouse Proximal Tubular Cells

Male C57BL/6 mice (6-8 weeks) were euthanized via intraperitoneal injection of sodium pentobarbital. The kidneys were dissected and transferred to Hank's salt solution. The kidney capsule was removed, and the renal cortex dissected and transferred to Hank's salt solution and minced. The minced cortex tissue was then digested in enzyme solution (1 ml Hank's salt solution with 0.75 mg collagenase and 0.75 mg trypsin inhibitor) for 60 min at 37°C. The cells were then mechanically separated from the digested tissue by being forced through a 40 *μ*m mesh. The cells were centrifuged at 50 × *g* for 2 min and washed with a culture medium. The cell suspension was transferred to a Percoll density gradient and centrifuged at 14,000 rpm for 1 h. The uppermost cells were the proximal tubular cells. Finally, the cells were seeded into six-well plates at a density of 5.0 × 10^5^ cells per well. Cell viability was determined using the trypan blue exclusion method. Confocal immunofluorescence was performed to detect primary mouse proximal tubular cells marker AQP1 (Supplemental Figure 1).

### 2.4. Serum Creatinine Quantification

Creatinine levels were evaluated in 30 *μ*l of serum from each mouse using the QuantiChromTM Creatinine Assay Kit (Bio Assay Systems, Hayward, CA, USA) following the manufacturer's instructions.

### 2.5. TUNEL Assay

Cell apoptosis was detected in paraffin-embedded kidney tissue sections using the commercially available TUNEL assays kit according to the manufacturer's instructions. Nuclei were stained with 4′,6-diamidino-2-phenylindole (DAPI). The number of TUNEL-positive cells in five random areas per slide was counted using an Olympus FV1000 confocal microscope. Apoptotic cells were identified as green fluorescent cells.

### 2.6. Flow Cytometry

The ratio of apoptotic cells to total cells was determined by flow cytometric analysis using an Annexin V-FITC/7-AAD kit (KGA1023-1026, Kaiji Biotechnology) according to the manufacturer's instructions.

### 2.7. Western Blot Analysis

Protein was extracted from primary proximal tubular cells or kidneys using a lysis buffer (RIPA P0013B, Beyotime Biotechnology) containing protease inhibitor phenylmethylsulfonyl fluoride (PMSF) and PPI. Aliquots of the protein samples (30 ∼ 80 *μ*g) were mixed with 5× loading buffer, separated on 10% (wt/vol) SDS/PAGE gels, and transferred to nitrocellulose membranes. Then, the membranes were blocked with 10% (wt/vol) nonfat skim milk at room temperature for 1 h and incubated with primary antibodies at 4°C overnight. The primary antibodies used were rabbit anti-Plin2 (1: 1000), rabbit anti-Bax (1 : 1000), rabbit anti-Bcl-2 (1 : 1000), rabbit anti-Caspase-3 (1 : 1000), rabbit anti-cleaved Caspase-3 (1 : 1000), rabbit anti-PPAR*α* (1 : 1000), and mouse anti–*β*-actin (1 : 1000). After three washes of 10 min each with Tris-buffered saline containing Tween 20 (TBS-T), the membranes were incubated for 1 h at room temperature with 1 : 10000 horseradish peroxidase- (HRP-) conjugated secondary antibodies. Detection of the bound antibody was carried out using a chemiluminescence substrate. Protein expression levels were quantified using ImageJ software.

### 2.8. Real-Time PCR

Total RNA was extracted from mouse tissues or primary mouse proximal tubular epithelial cells using TRIzol reagent (Sigma-Aldrich, T9424-200 ml). The RNA was reverse transcribed into complementary DNA (cDNA) using a PrimeScript RT Reagent Kit (TaKaRa, Japan) according to the manufacturer's instructions. Real-time PCR analysis was carried out using cDNA as template in the PCR reactions with SYBR Premix Ex Taq (TaKaRa). The PCR primer sequences are listed in Supplemental Table 1.

### 2.9. Cell Viability Assessment

Cell viability was determined using cell counting kit-8 (CCK-8) assays. Briefly, mouse tubular epithelial cells were grown to 70%-80% confluence in 96-well plates and then subjected to various treatments indicated. The CCK-8 regent was added to the medium and incubated for 1-4 h in the dark. Absorbance was measured at 450 nm using a microplate reader, and the cell viability was calculated.

### 2.10. Immunofluorescence

Frozen kidney sections were fixed with cold acetone for 10 min. The sections were permeabilized with 0.2% Triton X-100 for 10 min and then blocked with 5% bovine serum albumin for 30 min. The prepared sections were incubated with primary antibody at 4°C overnight. The sections were washed and then incubated with secondary antibody (Dye Light488-conjugated, green) for 30 min at room temperature. Nuclei were stained with DAPI. Images were acquired using the Olympus FV1000 confocal microscope.

### 2.11. Mitochondrial ROS Assay

Primary proximal tubular cell mitochondrial ROS was detected using MitoSOX Red Mitochondrial Superoxide Indicator (Invitrogen) following the manufacturer's instructions. Renal tissue ROS was detected in frozen sections using dihydroethidium (DHE) staining following the manufacturer's instruction. Nuclei were stained with DAPI solution. Images were acquired with confocal microscopy, and the fluorescent signal was quantified using ImageJ software.

### 2.12. Statistical Analysis

All data are expressed as mean ± S.E.M. and analyzed using the Prism software package (GraphPad Software). Unpaired two-sided Student's *t*-tests were used to differentiate the significance between two groups. Intergroup differences were analyzed using analysis of variance (ANOVA). A *P* value < 0.05 was considered statistically significant.

## 3. Results

### 3.1. Plin2 Is Upregulated in the Kidneys of Mice after I/R Treatment

**Figure 1:**
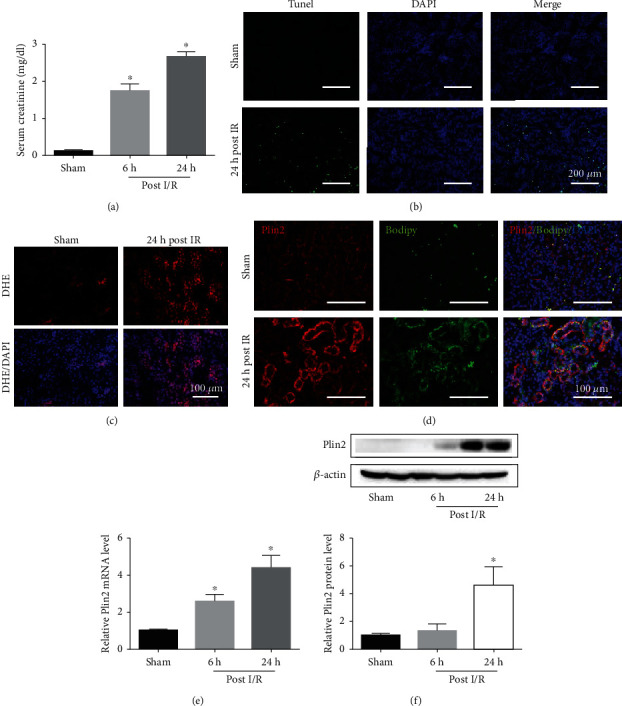
I/R-induced Plin2 upregulation and AKI. Eight-week-old C57/BL6 mice were treated with I/R and euthanized after 0, 6, and 24 h. (a) Serum creatinine (SCr) levels in the different groups of mice (*n* = 4-5). (b) Representative terminal deoxynucleotidyl transferase–mediated dUTP nick end-labeling (TUNEL) staining of kidney sections 24 h after renal IR (*n* = 5). (c) Representative images of dihydroethidium (DHE) staining of reactive oxygen species (ROS) generation of kidney sections 24 h after renal IR (*n* = 5). (d) Costaining for Plin2 and lipid droplet in kidney sections 24 h after renal IR (*n* = 5). (e) Quantitative RT-PCR analysis of Plin2 mRNA expression in the different groups of mice after renal IR (*n* = 5). (f) Western blot analysis of Plin2 expression in the different groups of mice after renal IR (*n* = 3). ^∗^*P* < 0.05 vs. sham mice. Data are presented as mean ± SEM. h: hour; IR: ischemia reperfusion.

To investigate changes in the context of I/R-induced AKI, mice treated with I/R were examined at different timepoints posttreatment (0, 6, and 24 h). Our results showed that I/R robustly induced serum creatinine levels, which were increased at 24 h (Figure 1(a)). Consistently, periodic acid–Schiff staining showed severe tubular and interstitial damage at 24 h (Supplemental Figure 2a). Furthermore, TUNEL staining demonstrated that the number of apoptotic cells increased significantly at 24 h after I/R (Figure 1(b)). In addition, mRNA expression of the proinflammatory mediators *IL-6* and *TNFα* increased after I/R treatment (Supplemental Figure 2b and 2c). Interestingly, the expression of IL-6 increased significantly at 6 h, whereas the expression of TNF*α* increased significantly at 24 h.

It has been indicated that the production of ROS remains high for 24 h after renal IRI [14]. Therefore, we used DHE staining to assess the production of ROS after renal IRI and found higher production (Figure 1(c)). It has been demonstrated that the production of ROS increases Plin2 expression in HepG2 cells [7]. To assess Plin2 expression after renal IRI, we analyzed tissue from I/R-induced AKI mice sacrificed 0, 6, and 24 h after I/R injury. Costaining for lipid droplet surface protein Plin2 and renal proximal tubular marker LTL in the kidneys indicated that Plin2 is mainly located in renal proximal tubulars (Supplemental Figure 2d). Costaining for Plin2 and BODIPY in the kidneys further demonstrated the levels of lipid droplet surface protein Plin2 increased after I/R (Figure 1(d)). RT-PCR showed the level of mRNA expression of Plin2 was robustly increased at 24 h, and western blotting demonstrated the protein levels also increased at 24 h (Figures 1(e) and 1(f)). These results indicated that Plin2 was upregulated after I/R-induced AKI. We presumed that the production of ROS and expression of Plin2 in kidney injury were interrelated.

### 3.2. Hydrogen Peroxide Treatment Increases Plin2 Expression in Primary Renal Proximal Tubular Cells

**Figure 2:**
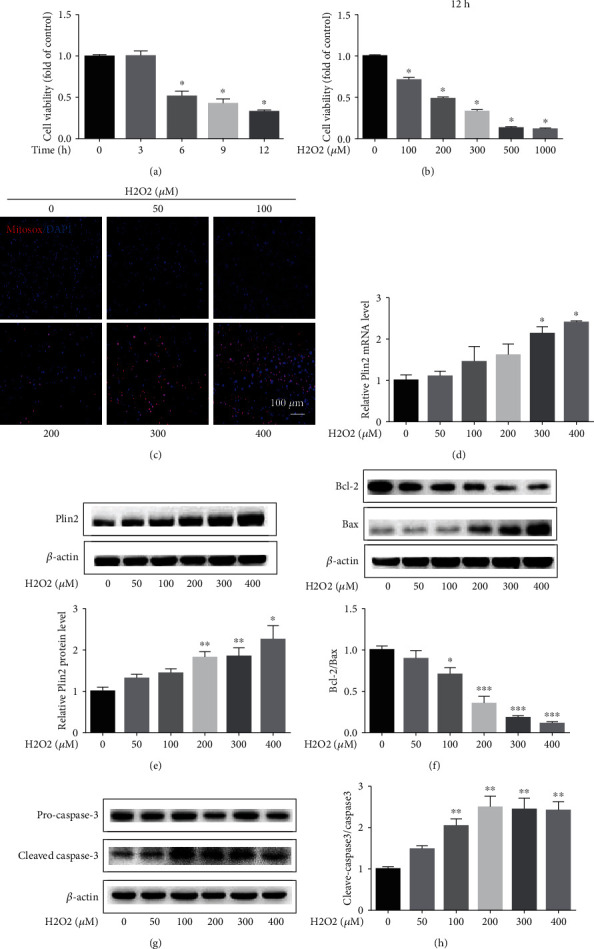
Plin2 levels increase after hydrogen peroxide treatment in primary renal proximal tubular cells. Primary renal proximal tubular cells were treated with hydrogen peroxide. (a, b) Cell viability measured using CCK-8 assays after hydrogen peroxide treatment (*n* = 10). ^∗^*P* < 0.05 vs. 0 h or 0 *μ*M hydrogen peroxide. (c) Images of mitochondrial reactive oxygen species (ROS) generation after hydrogen peroxide treatment for 12 hours. (d) Quantitative RT-PCR analysis of Plin2 mRNA expression after hydrogen peroxide treatment for 12 hours (*n* = 4). ^∗^*P* < 0.05 vs. 0 *μ*M hydrogen peroxide. (e–h) Western blot analysis of Plin2, BAX, Bcl-2, and cleaved caspase-3 expressions after hydrogen peroxide treatment for 12 hours (*n* = 4). ^∗^*P* < 0.05 vs. 0 *μ*M hydrogen peroxide; ^∗∗^*P* < 0.01 vs. 0 *μ*M hydrogen peroxide; and ^∗∗∗^*P* < 0.001 vs. 0 *μ*M hydrogen peroxide. Data are presented as mean ± SEM. H_2_O_2_: hydrogen peroxide.

We first evaluate the effect of hydrogen peroxide treatment on the viability of primary renal proximal tubular cells. Our results showed that cell viability significantly decreased in a dose-dependent and time-dependent manner upon exposure to hydrogen peroxide (Figures 2(a) and 2(b)), Supplemental Figure 3a–3c). As expected, hydrogen peroxide treatment significantly increased mitochondrial ROS generation in primary renal proximal tubular cells (Figure 2(c)). Plin2 expression was increased in a dose-dependent manner upon exposure to hydrogen peroxide treatment (Figures 2(d) and 2(e)). Furthermore, hydrogen peroxide treatment significantly increased apoptosis of primary renal proximal tubular cells in a dose-dependent manner (Figures 2(f)–2(h)).

### 3.3. Plin2 Knockdown Attenuates Apoptosis in Primary Renal Proximal Tubular Cells

**Figure 3:**
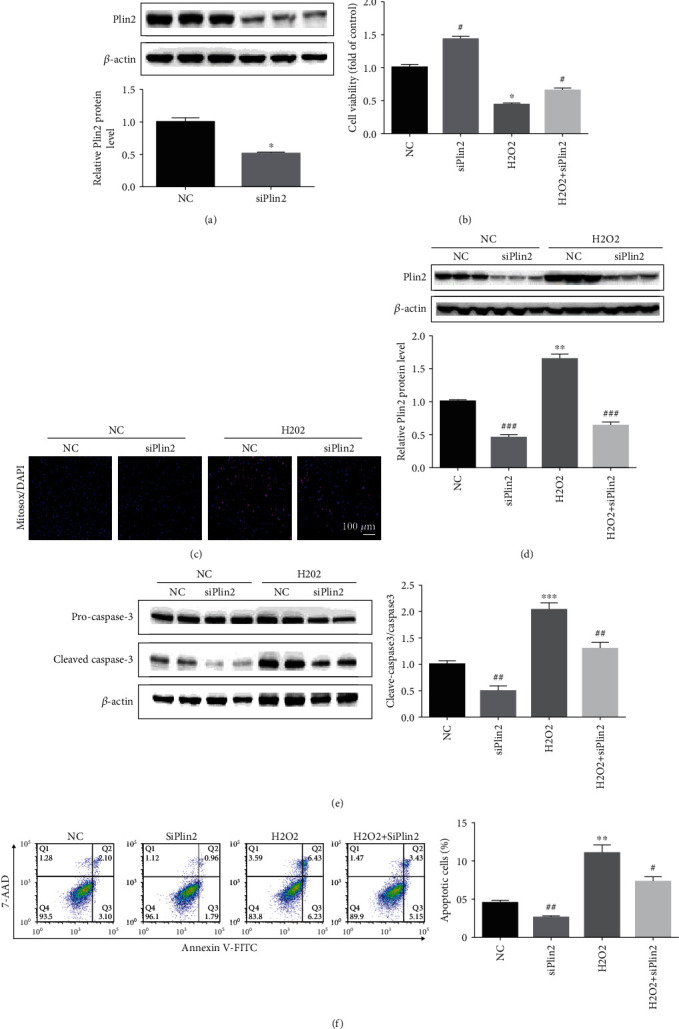
Knockdown of Plin2 attenuates apoptosis in primary renal proximal tubular cells. (a) Western blot analysis of Plin2 expression after Plin2 knockdown (*n* = 3). ^∗^*P* < 0.05 vs. the NC group. (b) Cell viability measured using CCK8 assays (*n* = 15). ^∗^*P* < 0.05 vs. the NC group, ^#^*P* < 0.05 vs. the NC or H_2_O_2_ group. Data are presented as mean ± SEM. (c) Images of mitochondrial reactive oxygen species (ROS) generation in PTECs transfected with Plin2 knockdown plasmid after hydrogen peroxide (300 *μ*M) treatment. (d, e) Western blot analysis of Plin2 (*n* = 3) and cleaved caspase-3 (*n* = 4) expression in primary renal proximal tubular cells transfected with Plin2 knockdown plasmid after hydrogen peroxide (300 *μ*M) treatment (*n* = 4). ^∗^*P* < 0.05 vs. the NC group, ^∗∗∗^*P* < 0.001 vs. the NC group, ^#^*P* < 0.05 vs. the NC or H_2_O_2_ group, and ^##^*P* < 0.01 vs. the NC or H_2_O_2_ group. (f) Cell apoptosis in hydrogen peroxide (300 *μ*M)-treated renal proximal tubular cells transfected with Plin2 knockdown plasmid or control plasmid. Annexin V-positive cells were considered apoptotic cells (*n* = 4). ^∗∗^*P* < 0.05 vs. the NC group, ^#^*P* < 0.05 vs. the H_2_O_2_ group, and ^##^*P* < 0.01 vs. the NC group. Data represent the mean ± SEM. H_2_O_2_: hydrogen peroxide; NC: negative control.

To further investigate the role of Plin2 in apoptosis, its expression was knocked down in primary renal proximal tubular cells (Figure 3(a)). Plin2 knockdown promoted cell viability and significantly decreased mitochondrial ROS generation upon exposure to hydrogen peroxide (Figures 3(b)–3(d)). In addition, this blocked the hydrogen peroxide-induced apoptosis in renal proximal tubular cells (Figures 3(e) and 3(f)). These results indicated that the knockdown of Plin2 could inhibit mitochondrial ROS generation and conferred protective effects against apoptosis in primary renal proximal tubular cells.

### 3.4. Plin2 Impacts Apoptosis via the Regulation of PPAR*α*

**Figure 4:**
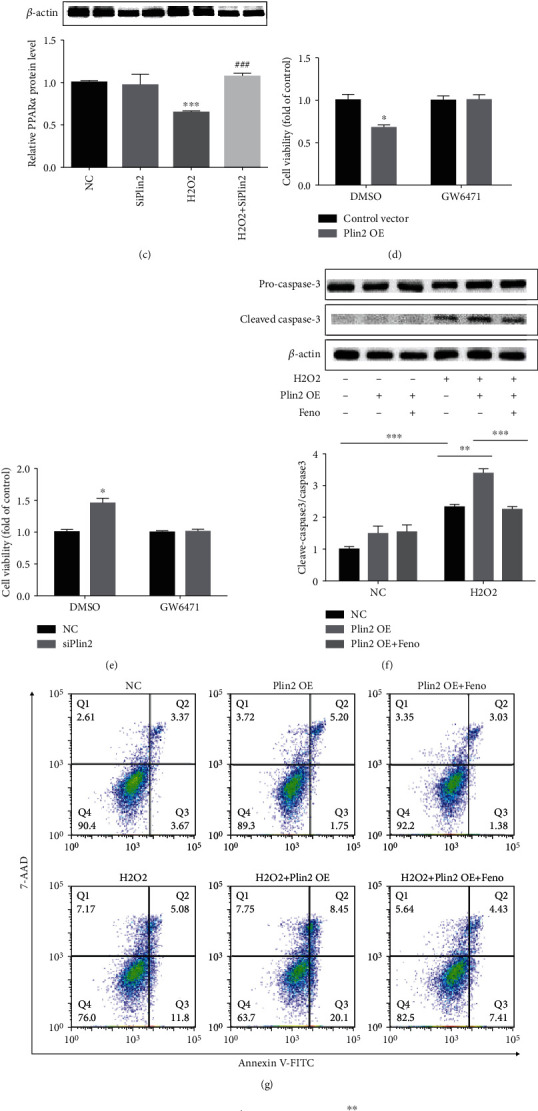
Plin2 impacts apoptosis via the regulation of PPAR*α*. (a, b) Expression of PPAR*α* after hydrogen peroxide treatment (*n* = 4). ^∗^*P* < 0.05 vs. 0 *μ*M hydrogen peroxide; ^∗∗^*P* < 0.01 vs. 0 *μ*M hydrogen peroxide; and ^∗∗∗^*P* < 0.001 vs. 0 *μ*M hydrogen peroxide. (c) Western blot analysis of PPAR*α* in primary renal proximal tubular cells transfected with Plin2 knockdown plasmids after hydrogen peroxide treatment (*n* = 4). ^∗∗∗^*P* < 0.001 vs. NC;^###^*P* < 0.001 vs. hydrogen peroxide. (d, e) Cell viability of Plin2 knockdown or overexpression in primary renal proximal tubular cells after hydrogen peroxide treatment (*n* = 4). ^∗^*P* < 0.05 vs. DMSO NC. (f) Western blot analysis of cleaved caspase-3 expression in primary renal proximal tubular cells transfected with Plin2 overexpression plasmid with or without fenofibrate after hydrogen peroxide (300 *μ*M) treatment (*n* = 4), ^∗∗^*P* < 0.01, ^∗∗∗^*P* < 0.001. (g, h) Cell apoptosis in hydrogen peroxide (300 *μ*M)-treated renal proximal tubular cells transfected with Plin2 knockdown plasmid or control plasmid with or without fenofibrate. Annexin V-positive cells were considered apoptotic cells (*n* = 3), ^∗^*P* < 0.05, ^∗∗^*P* < 0.01. Data represent mean ± SEM. H_2_O_2_: hydrogen peroxide; NC: negative control; OE: overexpression.

PPAR*α* is highly expressed in the proximal tubules and participates in the occurrence and development of kidney diseases [8–10]. It has been reported that PPAR*α* promotes the repair of kidney injury induced by I/R. We found that PPAR*α* expression decreased in a dose-dependent manner in renal proximal tubular cells treated with hydrogen peroxide (Figures 4(a) and 4(b)). Plin2 downregulation was accompanied by an increase in PPAR*α* expression under hydrogen peroxide treatment conditions (Figure 4(c)). Consistently, CCK-8 assays confirmed that the inactivation of PPAR*α* reversed the effects of Plin2 on renal proximal tubular cells (Figures 4(d) and 4(e)). Moreover, PPAR*α* activation reversed the apoptosis induced by Plin2 overexpression upon hydrogen peroxide treatment (Figures 4(f)–4(h)). Taken together, our data indicated that Plin2 downregulation alleviates renal proximal tubular cell apoptosis via the upregulation of PPAR*α*.

## 4. Discussion

**Figure 5:**
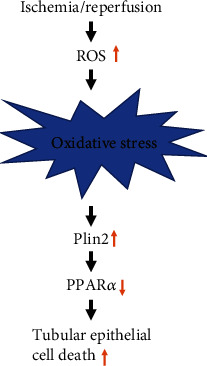
Proposed schema of the pathway for I/R-induced AKI, involving the promotion of mitochondrial reactive oxygen species (ROS) generation, upregulation of Plin2, and downregulation of PPAR*α*, resulting in cell apoptosis.

In the present study, we determined the relationship between Plin2 and PPAR*α* in the regulation of apoptosis in a model of AKI induced by I/R (Figure 5). Renal IRI resulted in the upregulation of Plin2, which inhibited PPAR*α* expression and increased mitochondrial ROS production, leading to cell apoptosis and AKI. Oxidative stress-induced injury is an important part of renal IRI [15]. The knockdown of Plin2 alleviated mitochondrial ROS-induced apoptosis in primary proximal renal tubular epithelial cells, which was mediated by the inhibition of PPAR*α* expression. Therefore, Plin2 knockdown may be targeted for the treatment of AKI induced by I/R.

Our results indicated that Plin2 was upregulated by ROS production in renal IRI. Perilipins are lipid droplet surface proteins that include five family members (Plin1–5) with different distributions and functions. Among them, Plin2 was the first identified and is highly expressed in adipose tissue and skeletal muscle of rodents and humans [16]. Plin2 is involved in the differentiation and lipolysis of adipose tissue, but its role in skeletal muscle is not yet clear. It plays an important role in the formation of lipid droplets in HepG2 cells, and Plin2 knockdown significantly reduces the size and number of lipid droplets [17]. However, its function and expression in the kidney was previously not well known. In the current study, we found that Plin2 was expressed in the normal kidney, mainly in the renal tubules. A previous study demonstrated that Plin2 is upregulated by ROS in HepG2 cells [7]. In the present study, we found that Plin2 was upregulated by hydrogen peroxide in primary proximal tubular epithelial cells, while Plin2 knockdown could reduce the production of ROS in tubular cells.

It is noteworthy that Plin2 is a lipid droplet surface protein and its protein structure and subcellular localization are highly conserved among different species, which is essential for the formation and morphological stability of lipid droplets [18]. Whether ROS affects the formation of lipid droplets in renal tubular cells was not explored in this study. A recent study also indicated that ROS affects not only the expression of Plin2 but also the formation of lipid droplets in hepatocytes [7]. Further studies are needed to verify the effect of ROS on Plin2 expression and the formation of lipid droplets in renal tubular cells. Whether Plin2 affects the formation of lipid droplets in the presence of ROS also needs to be addressed.

It has been demonstrated that Plin2 affects the PPAR*α*-RXRA and CREB-CREBBP pathways [7]. PPAR*α* is a member of the nuclear receptor superfamily, which is highly expressed in the heart, liver, kidney, muscle, and other tissues with abundant mitochondria and fatty acid betaoxidation [19, 20]. It plays an important role in maintaining the homeostasis of lipid metabolism. In the kidney, PPAR*α* is highly expressed in the proximal tubules and the ascending branch of the medullary loop, suggesting that it might be involved in the occurrence and development of a several kidney diseases, including cystic kidney disease 8 [21], alcoholic kidney injury [22], and diabetic kidney disease [23],. In addition, some evidence suggests that PPAR*α* promotes repair of the injured kidney induced by I/R [24, 25]. In our study, we found that the PPAR*α* expression patterns were opposite to those of Plin2 upon I/R or in primary proximal tubular epithelial cells treated with hydrogen peroxide. Plin2 thus has the potential to negatively regulate the expression of PPAR*α*, thereby regulating the production of ROS induced by I/R or hydrogen peroxide and affecting the apoptosis of renal tubular cells. In our study, we used the PPAR*α* agonist pioglitazone to pretreat primary renal tubular epithelial cells with Plin2 overexpression before hydrogen peroxide treatment. Pretreatment with pioglitazone abolished the apoptosis induced by Plin2 overexpression.

In addition, under hydrogen peroxide treatment conditions, pretreatment with the PPAR*α* agonist fenofibrate alleviated the decrease in cell viability induced by Plin2 overexpression, whereas the PPAR*α* inhibitor GW6471 inhibited the increase in cell viability induced by Plin2 knockdown. Collectively, our results provide insights suggesting that Plin2 increases apoptosis of renal tubular epithelial cells by inhibiting PPAR*α* expression and ROS produced upon renal I/R or hydrogen peroxide exposure.

Evidence indicates that Plin2 is an important player in renal tumors [10–12, 26]. Based on our experiments, we speculate that the expression of Plin2 is upregulated, whereas the expression of PPAR*α* is downregulated, in patients with AKI. Whether Plin2 participates in the development of AKI requires further investigation in the future.

In general, we illustrated that ROS production and the expression of Plin2 were significantly upregulated after renal I/R. The expression of Plin2 was also significantly increased after hydrogen peroxide treatment in vitro. The overexpression of Plin2 markedly increased apoptosis of proximal tubular epithelial cells after hydrogen peroxide treatment by inhibiting the expression of PPAR*α*. These results indicate, for the first time, that the targeted inhibition of Plin2 has a protective effect on ROS, which implies this is a potential target for the treatment of AKI.

## Data Availability

The figures and table used to support the findings of this study are included within the article and the supplementary information file.

## Supplementary Materials

Supplementary Materials Supplemental Figure 1: confocal immunofluorescence was performed to detect primary mouse proximal tubular cells marker AQP1. Supplemental Figure 2: I/R-induced AKI and inflammation. Supplemental Figure 3: cell viability decreases after hydrogen peroxide treatment in primary renal proximal tubular cells. Supplemental Table 1: primers used for amplifying mouse genes.
