# Supplementary material for: Perilipin 2 Impacts Acute Kidney Injury via Regulation of PPARα
Source: J Immunol Res. 2021 Sep 9;2021:9972704. doi: 10.1155/2021/9972704 (PMC8445733; doi:10.1155/2021/9972704)
Supplement: Supplementary Materials — Supplemental Figure 1: confocal immunofluorescence was performed to detect primary mouse proximal tubular cells marker AQP1. Supplemental Figure 2: I/R-induced AKI and inflammation. Supplemental Figure 3: cell viability decreases after hydrogen peroxide treatment in primary renal proximal tubular cells. Supplemental Table 1: primers used for amplifying mouse genes. [file 9972704.f1.docx]

**Supplemental Information**

**Supplemental Figure Legends**


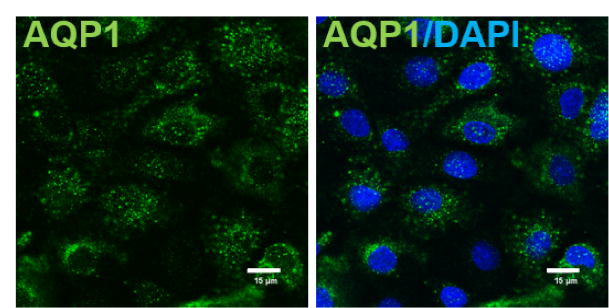


**Supplemental Figure 1.** **Confocal immunofluorescence was performed to detect primary mouse proximal tubular cells marker AQP1.**


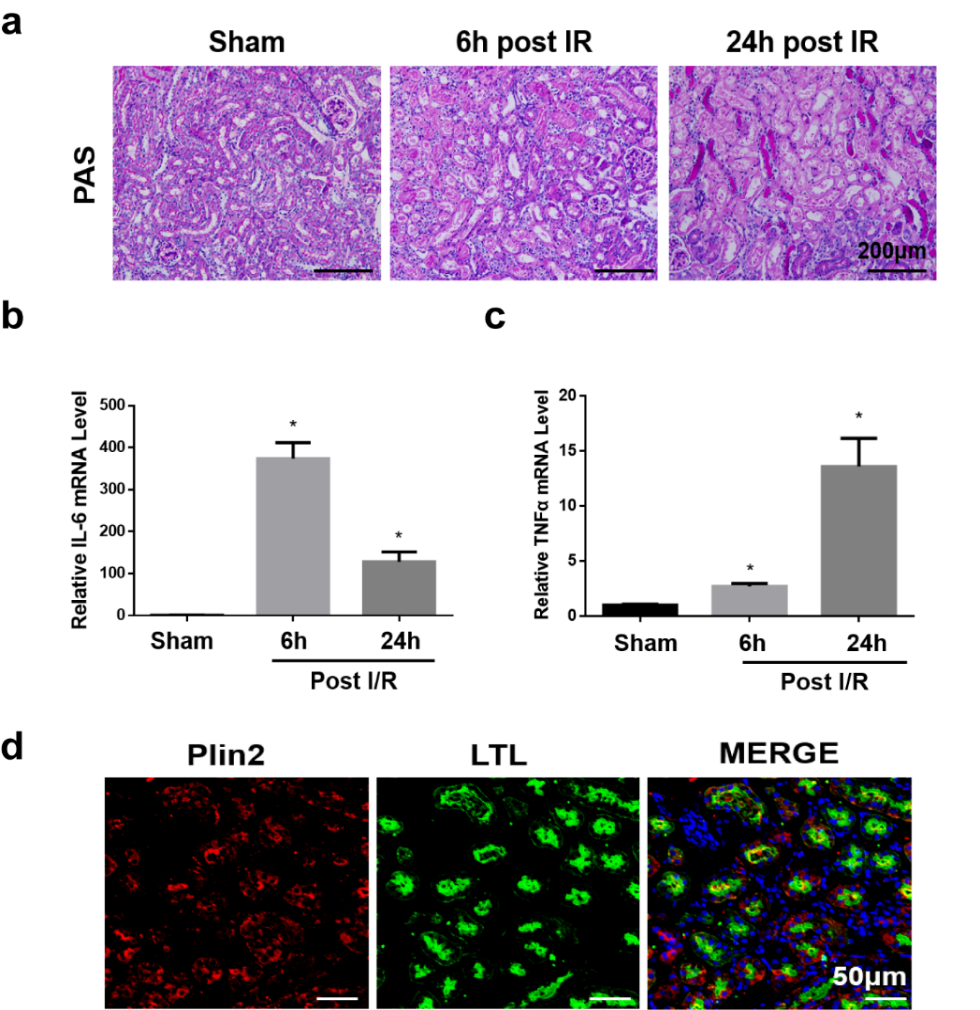


**Supplemental Figure 2. I/R-induced AKI and inflammation**. Eight-week-old C57/BL6 wild-type mice were treated with I/R and euthanized after 0, 6 and 24 hours. (a) Representative images of periodic acid–Schiff (PAS) staining of kidneys after renal IR (n=5). (b) Quantitative RT-PCR analysis of IL6、TNFα mRNA expression in the different groups of mice after renal IR (n=5). (c) Co-staining for Plin2 and proximal tubular marker in the kidneys. Plin2 (red) and proximal tubule, (LTL). * P < 0.05 vs. Sham mice. Data are presented as mean ± SEM.


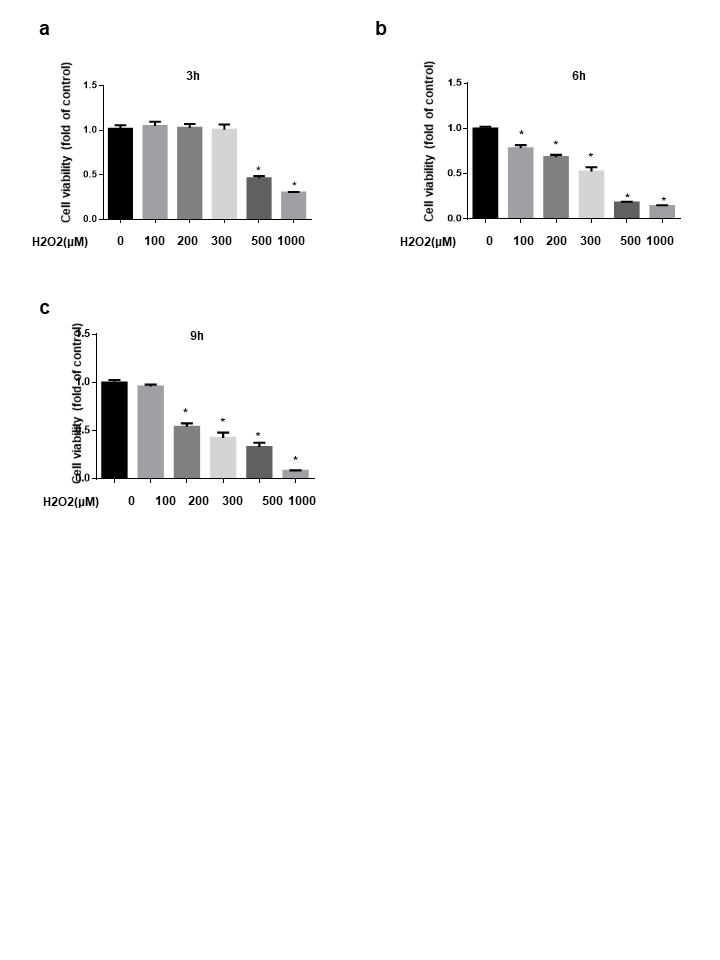


**Supplemental Figure 3. Cell viability decreased after hydrogen peroxide treatment in primary renal proximal tubular cells.** Primary renal proximal tubular cells were treated with hydrogen peroxide. (a-c) Cell viability measured by CCK8 after hydrogen peroxide treatment(n=9). * P < 0.05 vs. 0μM hydrogen peroxide. Data are presented as mean ± SEM.

**Supplemental Table 1. Primers used for amplifying mouse genes.**

| **Gene Name** | **Forward (5′-3′)** | **Reverse (5′-3)** |
| --- | --- | --- |
| **β-actin** | **AGCCATGTACGTAGCCATCC** | **GCTGTGGTGGTGAAGCTGTA** |
| **TNF-a** | **CATGAGCACAGAAAGCATGATCCG** | **AAGCAGGAATGAGAAGAGGCTGAG** |
| **IL-6** | **TAGTCCTTCCTACCCCAATTTCC** | **TTGGTCCTTAGCCACTCCTTC** |
| **PPARα** | **TGGGTACCAGGGAGAGACTG** | **CGGAAGAAACCTTTGCAGCC** |
| **Plin2** | **TCCACTGTCCACCTGATTGA** | **TGGCATGTAGTCTGGAGCTG** |
